# Proteomic Markers in the Muscles and Brain of Pigs Recovered from Hemorrhagic Stroke

**DOI:** 10.3390/genes13122204

**Published:** 2022-11-24

**Authors:** Liliya Fedulova, Ekaterina Vasilevskaya, Olga Tikhonova, Laura Kazieva, Galina Tolmacheva, Alexandr Makarenko

**Affiliations:** 1 V.M. Gorbatov Federal Research Center for Food Systems, Russian Academy of Sciences, 109316 Moscow, Russia; 2 Institute of Biomedical Chemistry, 119121 Mosow, Russia

**Keywords:** proteomics, intracerebral hematoma, protein–protein network interaction, bioinformatics

## Abstract

(1) Background: Stroke is the leading cause of serious long-term disability. Walking dysfunction and paresis of the upper extremities occurs in more than 80% of people who have had a stroke. (2) Methods: We studied post-genomic markers in biosamples of muscle and brain tissue from animals that underwent intracerebral hematoma and recovered after 42 days. Our purpose was to understand the biological mechanisms associated with recovery from hemorrhagic stroke. We analyzed the peptides formed after trypsinolysis of samples by HPLC-MS, and the results were processed by bioinformatics methods, including the establishment of biochemical relationships (gene to gene) using topological omics databases such as Reactome and KEGG. (3) Results: In the pig brain, unique compounds were identified which are expressed during the recovery period after traumatic injury. These are molecular factors of activated microglia, and they contribute to the functional recovery of neurons and reduce instances of hematoma, edema, and oxidative stress. Complexes of the main binding factors of the neurotrophins involved in the differentiation and survival of nerve cells were found in muscles. (4) Conclusions: A network of gene interactions has been constructed for proteins involved in the regulation of synaptic transmission, in particular presynaptic vesicular and endocytic processes. The presence of transmitters and transporters associated with stimulation of NMDA receptors at neuromuscular junctions shows the relationship between upper motor neurons and neuromuscular junctions.

## 1. Introduction

Among the numerous consequences of stroke, movement disorders are the main cause of long-term disability in the adult population. Limb paralysis, gait dysfunction, and paresis of the upper extremities persist in more than 80% of people who have had a hemorrhagic stroke [1]. The prognosis of recovery from movement disorders is difficult, and is based on a subjective clinical assessment. Six months after a stroke, two-thirds of survivors are unable to perform daily activities, and often do not return to work [2].

It is known that brain damage accompanied by a large cascade of reactions, including damage to the upper motor neurons and their descending corticospinal tracts, leads to subsequent muscle weakness [1,3,4]. The greatest effectiveness of motor rehabilitation is achieved within a three-month time window, although recovery may continue at a slower pace in subsequent months and years [5].

At present, the interaction of many biological mechanisms that limit recovery, including the motor system, after a stroke remains poorly understood. Various biomodels have been used to understand the biochemical pathways that affect recovery time after stroke. The use of pigs as a model for studying stroke is due to the similarity of their neurophysiological processes with humans [6]. Using pigs as an example, we sought to apply an exploratory approach to identifying molecular pathways of recovery, including moto-sensory functions, after stroke, since they are not well understood. The study of postgenomic markers of the organism by proteomic methods reflects both rapid changes in the acute period of stroke and molecular pathways of recovery after stroke, and changes in the long term [7].

The aim of our study was to identify proteins and genes encoding their expression, as well as to establish biochemical relationships in order to understand the biological mechanisms associated with recovery after hemorrhagic stroke.

## 2. Materials and Methods

As objects of study, biosubstrates were used—fragments of the brain of female pigs (hybrids of the Vietnamese Pot-bellied × Wiesenau, n = 9, 280–300 days old and weighing 44.7 ± 4.3 kg), divided into control, sham-operated, and reconvalescents—which were recovered after reproduction of intracerebral hematoma.

**Figure 1:**
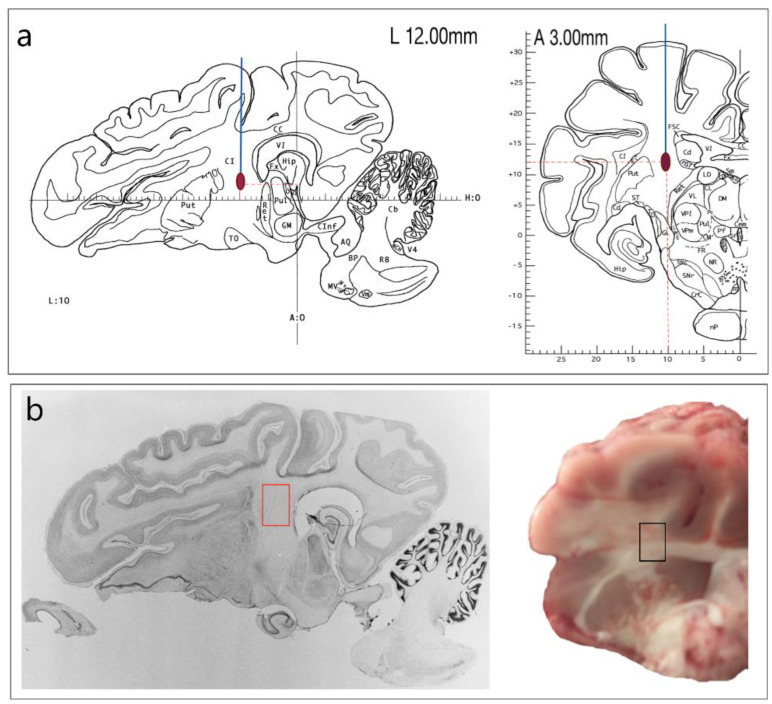
Model of intracerebral hemorrhage: (**a**)—stereoscopic model for simulating acute hemorrhagic stroke in the internal capsule [8]; (**b**)—location of brain sampling.

A model of left-sided intracerebral hemorrhage (IH) was used, stereotaxically (coordinates: A:3; H:10; L:12 [8]) inserted mandrel-wire knife destroyed appropriate brain structures (tissues and local blood vessels in the area of Capsule interna (CI)) for four rotations. Further autologous venous blood (from the ear vein of the same pig, 300 mkl) was injected into the damaged area (Figure 1a). This surgery allowed us to obtain a reproducible model of intracerebral hemorrhage with necessary precision, as was described in [9]. Sham surgery was performed without damaging the brain structures.

Full recovery after surgery was noted on day 42, according to the results of health monitoring (control of physiological parameters included body weight, physical activity, motor and cognitive functions, biochemicals, hematological blood parameters, distribution of leukocytes in the blood, and brain histology).

Brain samples (0.7 × 0.5 cm) were obtained from the left hemisphere in the IH zone (IP, ipsilateral) and right (CL, contralateral) hemispheres (Figure 1b). Similar samples were taken from 3 control (CON) and sham-operated (SHAM) pigs. Muscle tissue samples (biceps femoris) were also taken from IH and CON animals.

**Figure 2:**
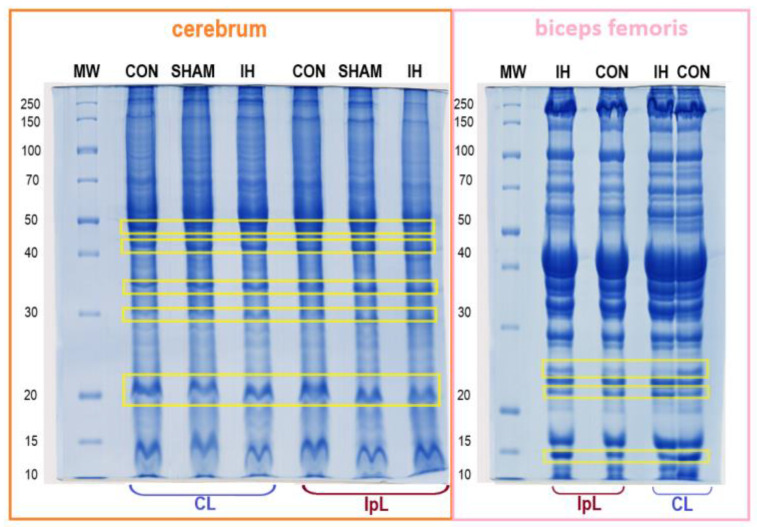
SDS-PAGE of pig brain and muscle biosubstrates: IH, convalescent pigs; SHAM, sham operated; CON, intact pigs; IpL, ipsilateral side; CL—contralateral side. Fractions selected for mass spectrometric studies are marked with yellow rectangles.

Figure 2 shows protein separation of the samples using sodium dodecyl sulfate-polyacrylamide gel electrophoresis (SDS-PAGE) [10]. For proteomic analysis, protein fractions were selected in the range of molecular weights of 50–40 kDa, 35–30 kDa, and 20 kDa for the brain and 25 kDa, 20 kDa, and 15 kDa for muscle tissues, because of the difference among the analyzed groups of samples. Thus, 5 protein fractions for brain samples and 3 protein fractions for muscle samples were selected for further proteomic analysis.

The HPLC-MS/MS analysis of the protein bands was carried out using equipment of the “Human Proteome” Core Facility Center (Institute of Biomedical Chemistry, Moscow). Protein hydrolysis with trypsin in a polyacrylamide gel was performed according to the protocol described earlier [11]. Peptides were analyzed using an Ultimate 3000 RSLCnano high-performance liquid chromatography (HPLC) system (Thermo Scientific, Waltham, MA, USA) connected to a Q-Exactive HF mass spectrometer (Thermo Scientific, USA), according to the procedure described in [12].

Proteins were identified by the MaxQuant v.1.6.17.0 program using the Andromeda search algorithm [13]. Proteins were identified using the Uniprot database, with a restriction on the species specificity of the studied organism Sus scrofa (UP8227 S_scrofa). The following search parameters were set: the cleaving enzyme trypsin, the accuracy of mass determination of monoisotopic peptides of ±5 ppm, the mass accuracy in tandem mass spectrometry (MS/MS) spectra of ±0.01 Da, and the possibility of skipping two trypsin cleavage sites. Oxidation of methionines and modification of cysteine by propionamide were considered possible modifications of the peptides. For validation of comparisons (pairing) of PSM (Peptide–Spectrum Matches) spectra and peptides, identification of peptides, and identification of proteins, an FDR (False Discovery Rate) value of no more than 0.01 (FDR < 0.01) was used. Proteins were considered to be reliably identified if at least two peptides were found for them. Label-free quantification of protein content was based on the empirical indicator Ibaq.

The PANTHER classification system was used to categorize and analyze the gene ontology (GO) of the identified proteins [14]. The Reactome pathways and reactions database (including data from NCBI, Ensembl, UniProt, and KEGG) were used to analyze the unique brain proteins of convalescent pigs [15]. Visual Paradigm (Hong Kong, China) was used for visualization.

The study was conducted in accordance with the Helsinki Declaration, and approved by the Ethics Committee of the V.M. Gorbatov Federal Research Center for Food Systems of the Russian Academy of Sciences (protocol #3/2019, dated 19 January 2019).

## 3. Results

**Figure 3:**
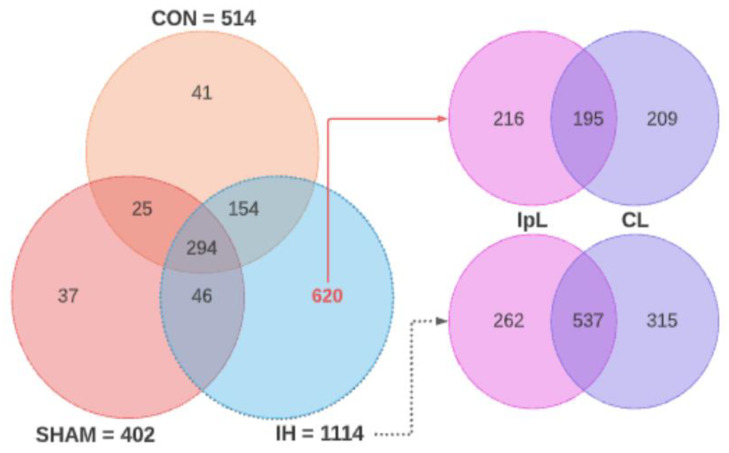
Venn diagrams showing the total number of porcine brain proteins identified and their distribution along the ipsilateral and contralateral sides for pigs with IH.

Proteomic analysis made it possible to identify a total of 514 compounds for control brain samples, 402 for sham-operated, and 1114 for IH. At the same time, 294 proteins were present in all samples; 25 were common for the control and SHAM, 154 for the control and IH, and 46 for SHAM and IH. A total of 41 proteins were identified only in control samples, and 37 proteins were identified only in SHAM samples. For the IH samples, 620 compounds not found in other samples were identified, of which 195 compounds were common to the ipsi- and contralateral hemispheres, 216 proteins were characteristic of the ipsilateral hemisphere, and 209 proteins were characteristic of the contralateral hemisphere (Figure 3).

In order to understand the function of the identified proteins, analyses of the GO and REACTOME pathways were performed. An analysis of the distribution of proteins by cellular components did not reveal any differences: 43–45% accounted for cellular anatomical entity (GO: 0110165: 44.92% of proteins for CON, 44.42% for SHAM, and 43.65% for IH); 15% for protein-containing complex (GO:0032991: 14.92%, 14.88%, and 14.88%, respectively); 40–41% for intracellular anatomical structure components (GO:0005622: 40.17%, 40.70%, and 41.47%, respectively).

**Figure 4:**
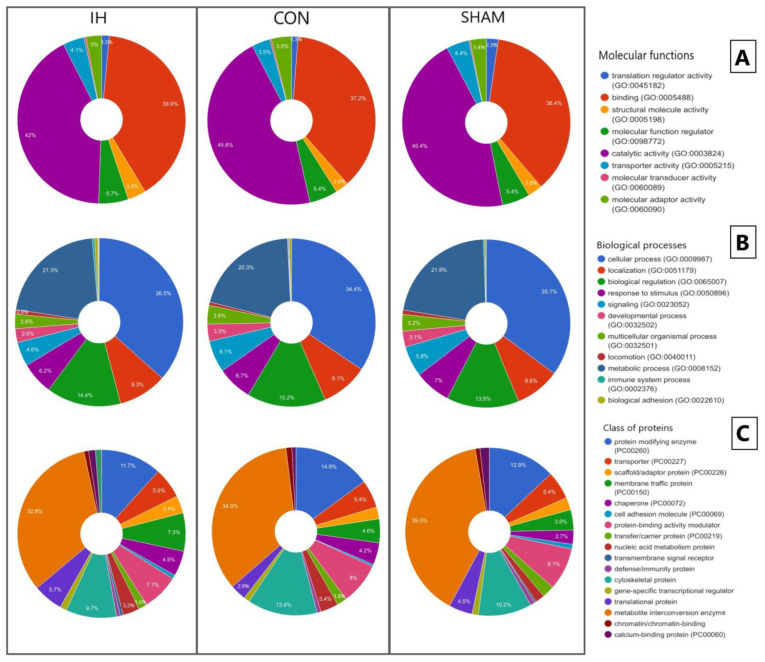
The PANTHER classification of the identified proteins in the brain by molecular function (**A**), biological processes (**B**), and class of proteins (**C**).

When distributing proteins according to molecular functions (Figure 4A), the main proportion of compounds in all samples belonged to proteins with binding and catalytic activities. In IH samples, compounds with molecular transducer activity and structural molecule activity increased. In samples IH and SHAM, relative to CON, a decrease in the amount of activity between the compounds and the translation regulator was revealed.

An analysis of the distribution of proteins by biological processes (Figure 4B) revealed that the main proportion of compounds in brain samples are involved in cellular, biological and metabolic processes, and localization. At the same time, the number of compounds involved in biological adhesion increased in the IH samples and immune processes. In IH samples, there was a decrease in the compounds involved in multi-organ processes, response to stimulus, signal processes, and developmental processes, which are processes occurring at the level of a multicellular organism. For IH samples, a unique protein, IGLL1 (GO:0044419), was identified.

When classifying the identified porcine brain compounds (Figure 4C), it was found that a significant proportion of the compounds belonged to metabolite interconversion enzymes, protein-modifying enzymes, cytoskeletal proteins, and protein-binding activity modulators. In IH samples, relative to CON and SHAM, an increase was found in the following: transport proteins, scaffold/adapter proteins, gene-specific transcription regulators, translational proteins, membrane transport proteins, chaperones, and transmembrane signaling receptors. At the same time, in IH samples, relative to CON and SHAM, a decrease in extracellular matrix proteins was detected by the intercellular signal molecule. In the IH and SHAM samples, relative to CON, the amount of nucleic acid binding proteins increased.

**Figure 5:**
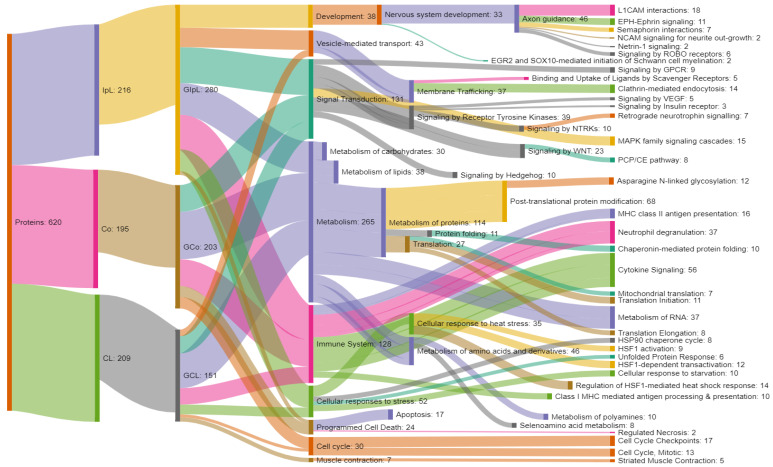
Genes of identified porcine brain proteins in the corresponding biological processes in the REACTOME: proteins—proteins unique for IH brain samples; IpL, proteins unique to the ipsilateral hemisphere; CL, proteins unique to the contralateral hemisphere; GIpL, co-proteins common to the ipsi- and contralateral hemispheres; and GCL, genes encoding the expression of proteins unique to the ipsilateral and contralateral hemispheres (Co are genes common to both hemispheres), for which a match was found in the REACTOME system.

The analysis of REACTOME pathways showed that 136 out of 184 genes corresponding to differentially expressed proteins characteristic of both hemispheres of the IH brain were associated with a total of 755 biological pathways (Figure 5). The most representative pathways included the following categories: cell cycle (checkpoint G2/M—16 genes, *p* = 8.3 × 10³;regulation of APC/C activators between G1/S and early anaphase—11 genes, *p* = 3.48 × 10³; G1/S checkpoint of DNA destruction—10 genes, *p* = 9.62 × 10³; ubiquitin-dependent degradation of cyclin D—9 genes, *p* = 9.38 × 10³), RNA metabolism (regulation of mRNA stability by gold-binding proteins—13 genes, *p* = 1.03 × 10³; AUF1 (hnRNP) mRNA binding and destabilization—10 genes, *p* = 9.45 × 10³), programmed cell death (18 genes, *p* = 9.74 × 10³; including apoptosis—17 genes, *p* = 2.6 × 10³), metabolism (regulation of ornithine decarboxylase—10 genes, *p* = 3.94 × 10³), signal transduction (degradation of GLI1 by the proteasome—10 genes, *p* = 2.43 × 10³; degradation of GLI2 by the proteasome—10 genes, *p* = 2.43 × 10³; conversion of GLI3 by the proteasome into GLI3R—10 genes, *p* = 2.43 × 10³), and immune system (cross-presentation of soluble exogenous antigens—9 genes, *p* = 8.02 × 10³).

Of those identified in the ipsilateral hemisphere, 165 out of 189 genes for differentially expressed proteins were associated with 813 biological pathways. The key factors were: biological development (axonal guidance—31 genes, *p* = 6.80 × 10³), cellular response to the stimulus (cellular response to heat stress—18 genes, *p* = 1.27 × 10³), protein metabolism (cooperation of prefoldin and TriC/CCT in actin and tubulin folding—9 genes, *p* = 1.26 × 10³; association of TriC/CCT with target proteins during biosynthesis—9 genes, *p* = 2.44 × 10³; cooperation of PDCL (PhLP1) and TRiC/CCT in the beta-fold of the G-protein—8 genes, *p* = 4.29 × 10³), signal transduction (neurotrophin retrograde signaling—7 genes, *p* = 2.33 × 10³; PCP/CE pathway—8 genes, *p* = 2.25 × 10³); vesicle-mediated transport (clathrin-mediated endocytosis, 14 genes, *p* = 4.57 × 10³; gap junction degradation, 5 genes, *p* = 1.78 × 10³); immune system (representation of MHC class II antigen—13 genes, *p* = 1.14 × 10³); and transport of small molecules (plasma clearance of lipoproteins—5 genes, *p* = 6.34 × 10³).

In the contralateral hemisphere of the brain, 199 genes for differentially expressed proteins were identified, of which 136 are associated with 529 biological processes. The most significant of these were processes related to the immune system (IL12—signaling pathway—12 genes, *p* = 7.1 × 10³), protein metabolism (initiation of eukaryotic translation—11 genes, *p* = 4.89 × 10³; termination of eukaryotic translation—8 genes, *p* = 2.10 × 10³; SRP-dependent co-translational protein targeted to the membrane—8 genes, *p* = 4.51 × 10³; mitochondrial translation—7 genes, *p* = 9.05 × 10³), cellular response to stimulus (EIF2AK4 (GCN2) response to amino acid deficiency—9 genes, *p* = 6.48 × 10³), metabolism (selenocysteine synthesis—8 genes, *p* = 3.03 × 10³), protein metabolism (peptide chain elongation—8 genes, 1.16 × 10³;), RNA metabolism (nonsense-mediated decay—8 genes, *p* = 5.89 × 10³), muscle contraction (striated muscle contraction—5 genes, 3.58 × 10³).

**Figure 6:**
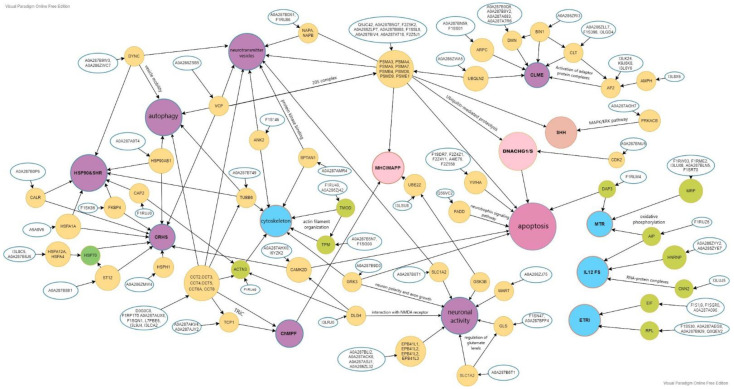
Unique proteins identified for the cerebral hemispheres of convalescent pigs according to biological responses: ChMPF—chaperonin-mediated protein folding; CLME, clathrin-mediated endocytosis; CRHS—cellular response to heat stress; HSP90 and SHR—HSP90 chaperone cycle for steroid hormone receptors with ligand present; MTR—mitochondrial translation; IL12 FS, interleukin 12 signaling pathway; ETRI translation initiation; MHCIMAPP—antigen processing and presentation mediated by MHC class I; SHH—Hedgehog signaling pathway; DNACHG1/S—G1/S DNA damage checkpoints.

Figure 6 compares the biological responses with the genes involved in them for the identified proteins unique to the brain of reconvalescent pigs.

Next, we carried out a comparative analysis of the protein composition of the muscle tissues of pigs IH and CON. In total, 201 compounds were identified for CON muscle samples, 219 for IH, and 152 proteins were present in all samples. There were 49 proteins identified only in control samples, and 66 proteins identified only in IH samples.

No differences were revealed in the samples with regard to the distribution of proteins by cellular components: cellular anatomical entity (GO:0110165) was found to account for 44.71% of the proteins in CON and 43.86% in IH; protein-containing complexes (GO:0032991) accounted for 16.86% and 16.14%, and intracellular components (GO:0005622) accounted for 40–41%; 38.43%, and 40.00%, respectively.

**Figure 7:**
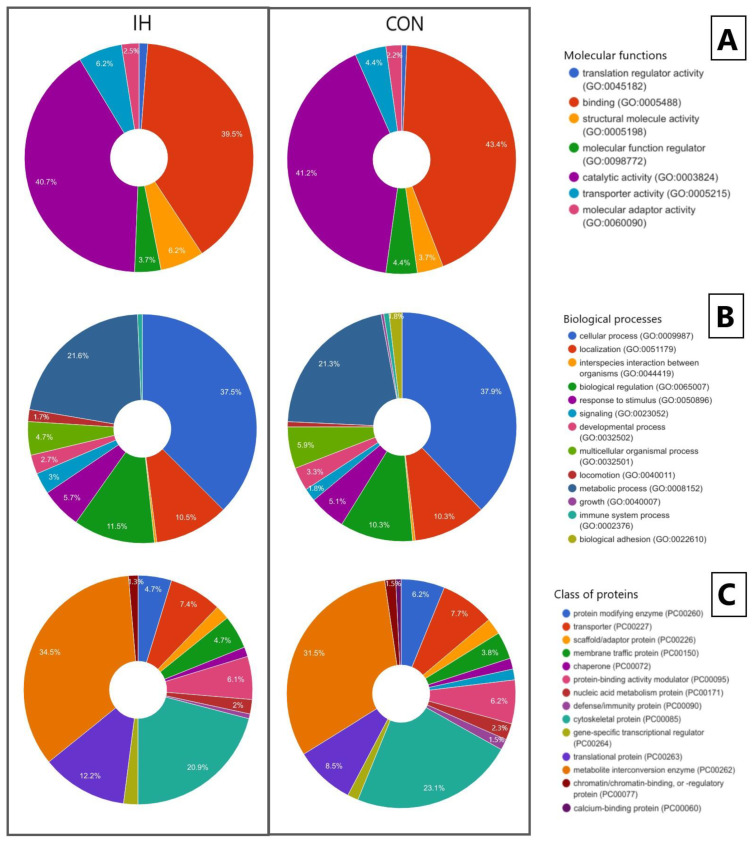
The PANTHER classification of the identified proteins in porcine muscle by molecular function (**A**), biological processes (**B**) and class of proteins (**C**).

According to molecular functions (Figure 7A), the main proportion of compounds in all samples belonged to proteins with binding and catalytic activities. In IH samples, compounds with molecular transduction activity and structural molecule function increased. In IH samples, relative to CON, a decrease in the number of compounds and translational regulators was revealed. When distributing proteins according to seven molecular functions, an increase was found in IH muscle samples relative to CON compounds, translational regulators, molecular adapter activity, structural function, and transport activity, with a decrease in molecular function regulator proteins.

According to biological functions (Figure 7B), an increase in IH was noted relative to CON compounds involved in biological and signaling processes, and reactions to irritants, with a decrease in proteins involved in developmental processes and multi-organ processes. In addition, the proteins involved in the processes of biological adhesion were found in CON, but were not detected in IH muscle samples.

An analysis of the distribution by 18 classes of identified protein compounds (Figure 7C) showed that in the muscle samples of IH pigs, relative to CON, an increase was found in gene-specific regulators of transcription, translation, and membrane transport, as well as a decrease in extracellular matrix proteins, scaffold/adapter proteins, intracellular signal molecules, and immune proteins.

Over the course of further study, the identified proteins of the muscle tissue of reconvalescent pigs were subjected to a comparative analysis in order to exclude compounds which were common with CON samples and proteins identified in all brain samples. They were also compared with data from the literature, which had been previously identified for the muscle tissues of pigs. Filtration revealed 13 proteins unique to IH muscles (Table 1).

The identified proteins are involved in the processes of mitochondrial translation (DR1, MRPL18), biogenesis, and functioning of organelles (MRPL18, TMEM11, CBFB, MRPL58). EIF4E is a signaling protein that regulates cell growth and proliferation [16], and H-FABP is a muscle-specific small heat shock protein.

An analysis of protein-coding genes revealed that 6 out of 13 genes encode proteins and control various processes, including cell adhesion, formation of intercellular connections and polarity of cells, intercellular and cell-matrix interactions, and regulating the path of MAPK and PLC. It is interesting to note here that the regulation of intracellular signaling cascades of these pathways may indirectly indicate that proteins (RAP1B; MSRB3; HSPB3; 4EBP1) are part of the complex of the main binding factors of the neurotrophins involved in the nerve cells’ differentiation and survival.

## 4. Discussion

In the brains of pigs which had recovered from intracerebral hematoma, unique proteins were identified to be involved in the biological reactions of proteostasis—autophagy (chaperonin-mediated and mitochondrial), protein biosynthesis (folding, formation of ensembles, and translocation of proteins), apoptosis, and endocytosis. Similar specific compounds expressed during recovery after traumatic injury may be molecular factors of activated microglia. In the acute period, activated microglia increases the production of pro-inflammatory, and potentially neurotoxic, mediators that damage neurons, and in the “secondary injury” phase, it promotes the resorption of hematoma and edema, reduces oxidative stress, and contributes to the functional recovery of neurons, which is reflected in the works of other researchers [17,18,19] and was indirectly confirmed in our experiment.

A significant portion of the IH proteins identified in the brain (>200) is involved in these processes, and modulates the folding, formation of ensembles, translocation of proteins, and protein quality control, maintaining cellular protein homeostasis. Thus, the identified proteins of the chaperone family (HSP60; Hsp70/HSPA1A; HSPA12A; HSP4; CALR/A0A287B0P6; HSP90AB1; TRIC/D0G0C8; F1RP17; A0A287AUX8; F1SQN1; L7PBE6; I3L9J4; I3LCA2) are responsible for the regulation of synthesized cellular processes, including novo proteins, refolding and degradation of misfolded proteins, disaggregation, membrane translocation, and endocytosis [15,20]. The components of the 20S proteasome complex (F1SSL6; A0A287BIV4; F2Z5K2; A0A287AT18; A0A287B088; A0A287B5Q7; A0A286ZLP7; Q5JC42) contribute to the removal of misfolded or damaged proteins [21,22].

The components of the translation initiation factor complex of the mRNA subset (F1RW03; F1RME2; I3LU08; A0A287BLN5; F1SRT0) are involved in cell proliferation, including the cell cycle, differentiation, and apoptosis [23,24]. The adapter protein complex (AP-2/I3LK24; K9J6K8; I3L6Y6) belongs to the components of the vesicle envelope, and is involved in clathrin-dependent endocytosis. Axonemal and cytoplasmic dyneins (A0A287B9W3; A0A286ZWC7) are involved in intracellular motility, including retrograde axonal transport, protein sorting, organelle movement, and spindle dynamics. Proteins which are regulators of neurotransmitter levels, including glutamate (GLS/F1SN47), as well as transporters/carriers of excitatory amino acids (SLC1A2/A0A287B6T1), have also been identified. The presence of multifunctional proteins (EPB41L/A0A287BLI2; A0A287ACK8; A0A287ASJ1; A0A286ZL32) was noted; these are able to bind with and stabilize dopamine receptors on the plasma membrane of neurons, impart stability and plasticity to the neuron membrane, and also affect the polarity of neurons and axon growth (A0A287B9D0). In addition, representatives of the family of cytoskeletal proteins (A0A287AMR4; A0A287B749; A0A286ZJ54; Q9N0Y9; F1RU49), essential scaffold proteins that also stabilize the plasma membrane and organize intracellular organelles, have been found.

The data obtained have been confirmed by earlier studies [9], where it was found that in reconvalescent pigs, after 24 h and 60 days, an increase in the expression of proteins involved in the prevention of unwanted protein aggregation and apoptosis, the release of neurotransmitters, and the assembly of the cytoskeleton was revealed.

The network of gene interactions which we have discovered can be considered in relation to proteins involved in the regulation of synaptic transmission, as well as in particular presynaptic vesicular and endocytic processes. The presence of transmitters and transporters indirectly indicates the secretion of glutamate precursors from synaptic vesicles and subsequent stimulation of NMDA receptors in neuromuscular synapses. Such processes are a humoral link between upper motor neurons and neuromuscular synapses [25].

Unique proteins identified in the muscle tissue of reconvalescent pigs are involved in several brain functions, including emotion, learning, and memory. Fatty acid binding proteins (FABPs) play a critical role as their cellular shuttles [26]. EIF4E is a signaling protein that regulates cell growth and proliferation, and can be activated in dendrites due to the activation of neurons [27]. MSRB3 is involved in protein–protein interactions, including protein folding, neuroprotection, and cell survival [28]. HSPB3 is a specialized chaperone involved in muscle cell differentiation, cytoskeletal remodeling, and protein degradation, and HSPB3 dysregulation causes neuromuscular diseases [29,30,31]. RAP1B has not been characterized, but the signaling pathway plays a dominant role in the control of intercellular and cell-matrix interactions, regulating the function of integrins, trophic factors, and other adhesion molecules [32]. Mitochondrial ribosomal proteins that we have identified in muscle tissue may be responsible for the degradation of polypeptides, induction of apoptosis, cells proliferation, and the reuse of broken ribosomes [33,34].

Together with the synaptic activation proteins identified in the brain, the detection of proteins in the brain and muscle tissues which are involved in the functioning and biogenesis of mitochondria, as well as signaling pathways of mitochondrial regulation, indicates the activity of mitochondria in distal axonal synapses and dendrites, which are key regulators of neuronal survival.

## 5. Conclusions

The signaling pathways which we identified mediate increased growth and branching of axons, an increase in the laminarity of neural structures and intercellular communications, a neuroprotective effect with increased remyelination, and an improvement in the functions of the motor cortex and its descending corticospinal tracts. The data obtained regarding the molecular mechanisms of recovery after hemorrhagic stroke can supplement our existing knowledge about molecular targets in the treatment, and can help to develop a prognostic system for assessing the recovery of patients after a stroke.

## Figures and Tables

**Table 1:** Unique proteins identified in the muscle tissue of convalescent pigs.

№	Uniprot ID	Protein	Γен	MW, kDa	Intensity
1	Q9BG57	Translation initiation factor 4E binding protein 1 (Fragment)	EIF4E	10,697	125,890,000
2	H6UI30	Heart fatty acid-binding protein	H-FABP	14,761	243,990,000
3	A0A287A271	SHSP domain-containing protein	HSPB3	16,808	189,480,000
4	A0A287B931	Uncharacterized protein	RAP1B	18,778	749,450,000
5	F1S534	Down-regulator of transcription 1 (Negative cofactor 2-beta)	DR1	19,245	15,008,000
6	A0A286ZNK1	Mitochondrial ribosomal protein L18	MRPL18	19,919	194,620,000
7	K7GP19	39S ribosomal protein L13, mitochondrial	MRPL13	2291	74,807,000
8	A0A286ZJR2	Mitochondrial ribosomal protein L58	MRPL58	23,503	35,632,000
9	A0A1Z1VUJ8	Peptide-methionine (R)-S-oxide reductase (EC 1.8.4.12)	MSRB3	19,931	56,872,000
10	A0A287B4D9	GTPase HRas isoform 1	HRAS	21,223	281,550,000
11	A0A287ARV8	Mitochondrial ribosomal protein L12	MRPL12	21,237	76,581,000
12	A0A286ZWB1	Transmembrane protein 11	TMEM11	21,287	128,060,000
13	M3V828	Core-binding factor subunit beta isoform X2	CBFB	21,478	69,361,000

## Data Availability

Not applicable.

## References

[1] Li S., Francisco G.E., Zhou P. (2018). Post-stroke Hemiplegic Gait: New Perspective and Insights. Front. Physiol..

[2] Conforto A.B., Machado A.G., Menezes I., Ribeiro N.H.V., Luccas R., Pires D.S., Leite C.C., Plow E.B., Cohen L.G. (2020). Treatment of Upper Limb Paresis with Repetitive Peripheral Nerve Sensory Stimulation and Motor Training: Study Protocol for a Randomized Controlled Trial. Front. Neurol..

[3] Rabek J.P., Hafer-Macko C.E., Amaning J.K., DeFord J.H., Dimayuga V.L., Madsen M.A., Macko R.F., Papaconstantinou J. (2009). A proteomics analysis of the effects of chronic hemiparetic stroke on troponin T expression in human vastus lateralis. J. Gerontol. Ser. A Biomed. Sci. Med. Sci..

[4] Beyaert C., Vasa R., Frykberg G.E. (2015). Gait post-stroke: Pathophysiology and rehabilitation strategies. Clin. Neurophysiol..

[5] Nguyen V.A., Riddell N., Crewther S.G., Faou P., Rajapaksha H., Howells D.W., Hankey G.J., Wijeratne T., Ma H., Davis S. (2020). Longitudinal Stroke Recovery Associated with Dysregulation of Complement System-A Proteomics Pathway Analysis. Front. Neurol..

[6] Hoffe B., Holahan M.R. (2019). The Use of Pigs as a Translational Model for Studying Neurodegenerative Diseases. Front. Physiol..

[7] Nguyen V.A., Carey L.M., Giummarra L., Faou P., Cooke I., Howells D.W., Tse T., Macaulay S.L., Ma H., Davis S.M. (2016). A Pathway Proteomic Profile of Ischemic Stroke Survivors Reveals Innate Immune Dysfunction in Association with Mild Symptoms of Depression—A Pilot Study. Front. Neurol..

[8] Félix B., Léger M., Albe-Fessard E.D., Marcilloux J.-C., Rampin O., Laplace J.-P., Duclos A., Fort F., Gougis S., Costa M. (1999). Stereotaxic atlas of the pig brain. Brain Res. Bull..

[9] Sidyakin A.A., Kaysheva A.L., Kopylov A.T., Lobanov A.V., Morozov S.G. (2018). Proteomic Analysis of Cerebral Cortex Extracts from Sus scrofa with Induced Hemorrhagic Stroke. J. Mol. Neurosci..

[10] Akhremko A., Fedulova L. (2021). Comparative study of weaning pigs’ muscle proteins using two-dimensional electrophoresis. Potravinarstvo. Slovak J. Food Sci..

[11] Zgoda V.G., Moshkovskii S.A., Ponomarenko E.A., Andreewski T.V., Kopylov A.T., Tikhonova O.V., Melnik S.A., Lisitsa A.V., Archakov A.I. (2009). Proteomics of mouse liver microsomes: Performance of different protein separation workflows for LC-MS/MS. Proteomics.

[12] Naryzhny S.N., Maynskova M.A., Zgoda V.G., Ronzhina N.L., Kleyst O.A., Vakhrushev I.V., Archakov A.I. (2016). Virtual-Experimental 2DE Approach in Chromosome-Centric Human Proteome Project. J. Proteome Res..

[13] Tyanova S., Temu T., Cox J. (2016). The MaxQuant computational platform for mass spectrometry-based shotgun proteomics. Nat. Protoc..

[14] Mi H., Muruganujan A., Thomas P.D. (2013). Large-scale gene function analysis with the PANTHER classification system. Nat. Protoc..

[15] Fabregat A., Sidiropoulos K., Viteri G., Marin-Garcia P., Ping P., Stein L., D’Eustachio P., Hermjakob H. (2018). Reactome diagram viewer: Data structures and strategies to boost performance. Bioinformatics.

[16] Qin X., Jiang B., Zhang Y. (2016). 4E-BP1, a multifactor regulated multifunctional protein. Cell Cycle.

[17] Bai Q., Xue M., Yong V.W. (2020). Microglia and macrophage phenotypes in intracerebral haemorrhage injury: Therapeutic opportunities. Brain.

[18] Duan X., Wen Z., Shen H., Shen M., Chen G. (2016). Intracerebral Hemorrhage, Oxidative Stress, and Antioxidant Therapy. Oxid. Med. Cell. Longev..

[19] Zambusi A., Ninkovic J. (2020). Regeneration of the central nervous system-principles from brain regeneration in adult zebrafish. World J. Stem Cells.

[20] Tittelmeier J., Nachman E., Nussbaum-Krammer C. (2020). Molecular Chaperones: A Double-Edged Sword in Neurodegenerative Diseases. Front. Aging Neurosci..

[21] Gan L., Cookson M.R., Petrucelli L., La Spada A.R. (2018). Converging pathways in neurodegeneration, from genetics to mechanisms. Nat. Neurosci..

[22] Klaips C.L., Jayaraj G.G., Hartl F.U. (2018). Pathways of cellular proteostasis in aging and disease. J. Cell Biol..

[23] Chen Y., Cao B., Zheng W., Sun Y., Xu T. (2022). eIF3k inhibits NF-κB signaling by targeting MyD88 for ATG5-mediated autophagic degradation in teleost fish. J. Biol. Chem..

[24] Shliapina V.L., Yurtaeva S.V., Dontsova O.V. (2021). At the Crossroads: Mechanisms of Apoptosis and Autophagy in Cell Life and Death. Acta Nat..

[25] Kasimov M.R., Fatkhrakhmanova M.R., Mukhutdinova K.A., Petrov A.M. (2017). Hydroxycholesterol enhances synaptic vesicle cycling in the mouse neuromuscular junction: Implication of glutamate NMDA receptors and nitric oxide. Neuropharmacology.

[26] Shioda N., Yamamoto Y., Watanabe M., Binas B., Owada Y., Fukunaga K. (2010). Heart-type fatty acid binding protein regulates dopamine D2 receptor function in mouse brain. J. Neurosci. Off. J. Soc. Neurosci..

[27] Moon I.S., Lee H.J., Park I.S. (2012). Dendritic eIF4E-binding protein 1 (eIF4E-BP1) mRNA is upregulated by neuronal activation. J. Korean Med. Sci..

[28] Sreekumar P.G., Hinton D.R., Kannan R. (2011). Methionine sulfoxide reductase A: Structure, function and role in ocular pathology. World J. Biol. Chem..

[29] Tiago T., Hummel B., Morelli F.F., Basile V., Vinet J., Galli V., Mediani L., Antoniani F., Pomella S., Cassandri M. (2021). Small heat-shock protein HSPB3 promotes myogenesis by regulating the lamin B receptor. Cell Death Dis..

[30] Vendredy L., Adriaenssens E., Timmerman V. (2020). Small heat shock proteins in neurodegenerative diseases. Cell Stress Chaperones.

[31] La Padula V., Staszewski O., Nestel S., Busch H., Boerries M., Roussa E., Prinz M., Krieglstein K. (2016). HSPB3 protein is expressed in motoneurons and induces their survival after lesion-induced degeneration. Exp. Neurol..

[32] Shah S., Brock E.J., Ji K., Mattingly R.R. (2019). Ras and Rap1: A tale of two GTPases. Semin. Cancer Biol..

[33] Cheong A., Archambault D., Degani R., Iverson E., Tremblay K.D., Mager J. (2020). Nuclear-encoded mitochondrial ribosomal proteins are required to initiate gastrulation. Development.

[34] Huang G., Li H., Zhang H. (2020). Abnormal Expression of Mitochondrial Ribosomal Proteins and Their Encoding Genes with Cell Apoptosis and Diseases. Int. J. Mol. Sci..

